# The Treatment of Insanity: Existing Defects and their Remedy

**Published:** 1907-04-27

**Authors:** 


### THE TREATMENT OF INSANITY.

### Existing Defects and their Remedy.

### III. THE LIFE IN ASYLUMS, HOSPITALS, AND LICENSED HOUSES.

The public asylum offers a refuge for the insane poor; the registered hospital and the licensed house are for the well-to-do. It is true that there are some large provincial licensed houses in which pauper patients are received, but generally the statement holds good. Whichever of the institutions is visited by a medical observer, he will find certain characters that are common to them all. He will find them pleasantly situated, with ornamental grounds, laid out in shrubberies and adorned in summer with abundance of flowers; he will see cricket grounds, bowling greens, lawn-tennis courts, skittle alleys, aviaries, and everything that makes grounds attractive. He will see a building that ranges, according to its age, from solid comfort to more or less gaudy magnificence, and, on entering, he may or may not be greeted by a peculiar and most unpleasant odour, the "asylum smell." Going into the wards, he will find, even in the pauper asylums, comfort, brightness, and a standard of furniture, fittings, decoration, light, space, and warmth that is far in excess of the standard that prevails in the homes of the great majority of the inmates; and in some of the registered hospitals and licensed houses he will find that comfort is merged into luxury, often of a very expensive character.

In either case he will find that a great deal of ingenuity has been expended on means and appliances for keeping the patients in safe custody, and for concealing, as far as possible, the means of doing so. He will find high walls deprived of their forbidding and prison-like character by being sunk for half their height in the ground. He will find the windows rendered impermeable to the human body, not by massive iron bars, but by a light framework of iron or steel into which the glass is fixed. He will find that a vast amount of care and ingenuity has been devoted to so modifying the structure and fittings of the building as to deprive the patients of opportunities for injuring themselves. Projections from which a noose could be suspended are carefully abolished. All doors are made to open outwards. The gas cannot be turned on without a special key. The bath taps are so arranged that the cold water must be turned on first. The fires are carefully guarded, and the guards are locked. The windows are made to open only just so much that, while plenty of air can get in, no one can get out. The same care is evident in everything. Knives are kept locked in a box, which again is locked in a cupboard, and are counted before and after each time of using. Brooms and brushes are locked up when not in use. Everything is under lock and key.

Now if he seeks to know how the lives of the inmates are ordered, he finds evidence of the same careful, systematic, and sedulous study. Recreation is cared for fully as much as occupation. Every asylum of any size has its band, its ball room, its theatre, and concert hall. Every ward has its bagatelle board, its cards, dominoes, draughts, chess and other games. There is a lending library. If the asylum is large enough to have its own chapel, as most asylums are, there is a choir which meets regularly for practice. Out of doors there are cricket, lawn tennis, bowling, fives, skittles, racquets, and what not. Every week there is a dance; concerts, theatrical performances, conjuring and variety entertainments are frequent. The lives of few people contain so many opportunities and invitations to recreation as do those of the inmates of institutions for the insane.

In other ways, too, the comfort and convenience of the inmates of asylums are studied. Private patients wear, of course, their own clothes, and, when the patient has not enough sense to care for himself, care is taken for him that he is clad sufficiently and properly for the time of the year. In the pauper asylums not only is the clothing good and sufficient, but appearance is studied, uniformity is avoided as far as possible, and opportunity and encouragement are given to the female patients to make such little additions in the way of ornament and embellishment as their means and taste may suggest. The food is abundant, of good quality, and varied; and extra delicacies for the sick are provided without stint.

To make the care of the insane thoroughly efficient, and to provide against the many contingencies in asylum life that are unknown in life outside asylums, it is very necessary that there should be in every institution a sufficient staff of attendants and nurses, thoroughly trained in, and adapted for, their peculiar duties. For such a staff careful provision is made. Their numbers are ample; their pay is good; pensions are provided for them when they are past work; they are taught and trained in an elaborate system of lectures and examinations by the medical staff of the asylum; and at the end of their curriculum they are examined by an independent authority, and diplomas are issued to those who pass the test.

That nothing may be wanting to render the lives of the insane as luxurious and as like to the lives of sane persons as possible, many of the registered hospitals and licensed houses have branch establishments at the seaside or in some health resort, to which those of the patients who are fit to go, can be sent when it seems that a change would benefit them.

In short, everything that can be done or devised to cheer the lives of the inmates of institutions for the insane, and to mitigate the irksomeness of their confinement is done liberally and lavishly; and if we regard institutions for the insane as places in which the inmates are to be made comfortable, to be luxuriously housed and fed and occupied and amused, we are compelled to testify that they serve their purpose with admirable completeness. But if we regard insane persons as sick persons afflicted with a grevious malady, and if we regard institutions for the insane as hospitals to which sick persons are sent to be treated for this grievous malady, and restored, if possible, to health, we must render a very different account of them.

